# Composite Lymphoma Comprising Extranodal NK/T-Cell Lymphoma and Diffuse Large B-Cell Lymphoma

**DOI:** 10.1155/2018/1583925

**Published:** 2018-10-31

**Authors:** Shin Nagai, Junji Hiraga, Noriyuki Suzuki, Naruko Suzuki, Yusuke Takagi, Michihiko Narita, Yoshitoyo Kagami

**Affiliations:** ^1^Department of Hematology, Toyota Kosei Hospital, Toyota, Japan; ^2^Department of Pathology, Toyota Kosei Hospital, Toyota, Japan

## Abstract

We report a rare case of composite lymphoma comprising extranodal NK/T-cell lymphoma, nasal type, (ENKL) and diffuse large B-cell lymphoma (DLBCL) in a 70-year-old man complaining of fatigue. Computed tomography showed multiple consolidations in both lungs, and ENKL was diagnosed from transbronchial lung biopsy. Positron emission tomography also detected abnormal uptake in the stomach, and DLBCL was diagnosed from subsequent gastroscopy. Two courses of chemotherapy including rituximab achieved reduction in DLBCL, but ENKL proved resistant to this treatment and progressed. Concomitant ENKL and DLBCL have not been previously described among reports of composite lymphomas.

## 1. Introduction

Composite lymphoma is defined as two or more morphologically and immunophenotypically distinct lymphomas arising in the same patient [1, 2]. This rare disease represents 1–4.7% of newly diagnosed malignant lymphomas [3]. Such cases usually comprised B-cell lymphoma and classical Hodgkin's lymphoma (CHL) [4–7] although some cases of B-cell lymphoma with T-cell lymphoma have been described [8, 9]. Extranodal NK/T-cell lymphoma, nasal type, (ENKL) is a refractory malignant lymphoma typically located in the nasal cavity, showing characteristics of highly aggressive development, resistance to therapies, and poor prognosis [10]. To the best of our knowledge, no previous reports have described composite lymphoma including ENKL. Herein, we report a case of composite lymphoma comprising ENKL and diffuse large B-cell lymphoma (DLBCL).

## 2. Case Presentation

**Figure 1:**
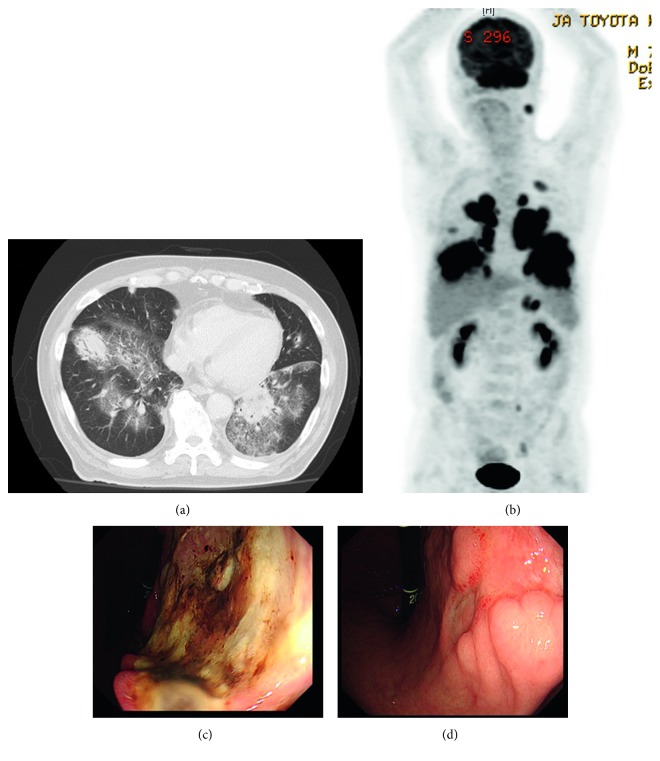
(a) CT of both lungs detects multifocal nodular lesions with ground-glass shadows. (b) PET detects abnormal uptake in multifocal lung lesions and the gastric body. (c) Before treatment, gastroscopy reveals an ulcerative lesion in the gastric body. (d) After treatment, gastroscopy shows improvement of the gastric lesion.

**Figure 2:**
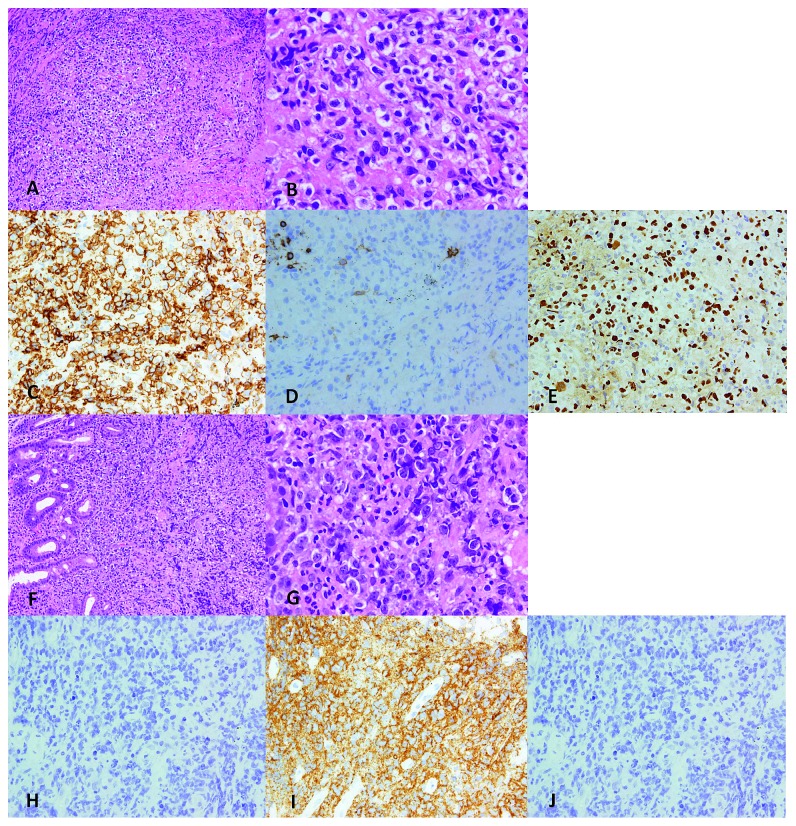
Pathological examinations of the lung (a–e) and gastric (f–j) lesions. (a, f) 100× magnification; (b, g) 400× magnification; other images 200× magnification. (a, b, f, g) Hematoxylin and eosin staining; (c–e, h–j) immunohistochemical staining. (a, b) Multiple lesions in the lungs show destruction of the pulmonary lobes and replacement with large tumor cells. (c) Tumor cells appear CD3-positive. (d) Tumor cells appear CD20-negative. (e) Tumor cells appear EBER-positive. (f, g) In the gastric lesion, tumor cells have infiltrated the mucosal epithelium. (h) Tumor cells appear CD3-negative. (i) Tumor cells appear CD20-positive. (j) Tumor cells appear EBER-negative.

The patient was a 70-year-old man who presented with fatigue and loss of appetite. He had a medical history of diabetes mellitus (DM) and hypertension and was receiving pharmacotherapy for both diseases. Laboratory examination showed thrombocytopenia (83,000/*µ*L; normal range: 140,000–400,000/*µ*L) and an elevated concentration of lactate dehydrogenase (LDH) (464 IU/L; normal range: 119–229 IU/L). DM was poorly controlled (hemoglobin A1c: 8.8%; normal range: 4.6–6.2%). Chest X-ray and computed tomography (CT) showed consolidation and surrounding ground-glass shadows in both lungs (Figure 1(a)), and transbronchial lung biopsy was therefore performed. Histopathological analysis revealed diffuse proliferation of medium-sized lymphoid cells. Tumor cells showed expressions of CD3, CD4, CD56, TIA-1, and granzyme B and in situ hybridization for Epstein–Barr virus- (EBV-) encoded small RNA (EBER-ISH), but absence of CD5, CD8, CD10, and CD20, leading to the diagnosis of ENKL (Figures 2–2). Otolaryngological examination was performed on a precautionary basis, but no abnormalities of the nasal mucosa were found. Positron emission tomography (PET)/CT was performed to search for other lesions, revealing abnormal uptake in the stomach in addition to the lung lesions (Figure 1(b)). Gastroscopy showed an ulcerative lesion (Figure 1(c)) that was biopsied. Histopathological analysis showed diffuse proliferation of large lymphoid cells infiltrating under the mucosa. Tumor cells lacked expressions of CD3, CD5, CD10, CD56, bcl2, bcl6, and EBER-ISH and positive results for CD20, CD79a, and MUM1, leading to the diagnosis of DLBCL (nongerminal center B-cell-like type) (Figures 2–2). Negative results were obtained for *Helicobacter pylori*. Bone marrow aspiration showed no invasion of tumor cells. The serous ferritin level was 2,260 ng/mL (normal range: 39.4–340 ng/mL). Antibodies to EBV showed a prior infection pattern, but EBV-DNA was elevated to 1.7 × 10^5^ copies/10^6^ cells and the concentration of soluble interleukin 2 receptor was 3,760 IU/mL (normal range: 145–519 IU/mL). We diagnosed composite lymphoma comprising ENKL and DLBCL. Chemotherapy was started with dexamethasone, etoposide, ifosfamide, and carboplatin (DeVIC) plus rituximab. During the clinical course, bone marrow was strongly suppressed and febrile neutropenia occurred. Piperacillin/tazobactam and granulocyte-colony stimulating factors were used, and platelet transfusions were necessary to address severe thrombocytopenia. After two courses of chemotherapy, gastrointestinal endoscopy showed shrinkage of the ulcerative lesion (Figure 1(d)) and elimination of lymphoid cells in the biopsy. On the other hand, lung lesions did not show any improvement, and the chemotherapy regimen was therefore changed. After one course of chemotherapy with gemcitabine, dexamethasone, and cisplatin (GDP), the disease remained progressive and dyspnea appeared. Best supportive care was initiated, and the patient died 3 months after diagnosis.

## 3. Discussion

We have reported a case of composite lymphoma comprising ENKL and DLBCL. To the best of our knowledge, composite lymphomas with ENKL have not been reported previously. Although we could not assess cell surface CD3 by flow cytometry as the specimen was obtained by transbronchial lung biopsy, cells were positive for CD56, EBER-ISH, and cytotoxic molecules. We therefore diagnosed the lung lesion as ENKL. According to the World Health Organization (WHO) criteria, peripheral T-cell lymphoma not otherwise specified (PTCL-NOS) is described in rare cases as EBV-positive and sometimes shows CD56 positivity [11]. To strictly divide these two pathologies (ENKL and PTCL-NOS), TCR rearrangements should be considered, but in this case, the lung biopsy specimens were too small to explore. An atypical etiology was suggested in this case, as the primary site was not the nasal cavity typically seen in ENKL [10]. The histopathological analysis of DLBCL was negative for EBER-ISH, differing from EBV-positive DLBCL. The clinical course thus did not match chronic active EBV infection. Immunodeficiency resulting from methotrexate treatment can cause lymphoma in methotrexate-related lymphoproliferative disease, and approximately 50% of such cases involve DLBCL, and 40% are positive for EBER-ISH [12]. In this case, poor control of DM and old age may have contributed to immunodeficiency and triggered EBV infection and composite lymphoma.

ENKL is a disease with poor prognosis. Longer survival can be expected with the combination of chemotherapy and radiation within the limited stage but may be unlikely in the advanced stage [10]. Although the SMILE regimen with dexamethasone, methotrexate, ifosfamide, L-asparaginase, and etoposide has been reported to offer a response rate of 79% and a complete response rate of 45% against advanced-staged ENKL among patients under 70 years old [13], we selected the DeVIC regimen due to the high age of the patient. We also administered rituximab against DLBCL. Although rituximab proved effective against DLBCL leading to the elimination of lymphoma cells from biopsy, no effect against ENKL was seen. Development of effective therapies against advanced ENKL in elderly patients is necessary.

Composite lymphoma is a rare disease, and only few cases have been reported. Clarification of the tumor characteristics and effective therapies will require accumulation of further reports.
